# Binding of Translationally Controlled Tumour Protein to the N-Terminal Domain of HDM2 Is Inhibited by Nutlin-3

**DOI:** 10.1371/journal.pone.0042642

**Published:** 2012-08-13

**Authors:** Garth Funston, Walter Goh, Siau Jia Wei, Quah Soo Tng, Christopher Brown, Loh Jiah Tong, Chandra Verma, David Lane, Farid Ghadessy

**Affiliations:** 1 School of Clinical Medicine, University of Cambridge, Addenbrookes Hospital, Cambridge, United Kingdom; 2 p53Lab (A*STAR), Singapore, Singapore; 3 Bioinformatics Institute (A*STAR), Singapore, Singapore; 4 School of Biological Sciences, Nanyang Technological University, Singapore, Singapore; 5 Department of Biological Sciences, National University of Singapore, Singapore, Singapore; Rush University Medical Center, United States of America

## Abstract

Translationally Controlled Tumour Protein (TCTP), a highly conserved protein present in all eukaryotic organisms, has a number of intracellular and extracellular functions including an anti-apoptotic role. TCTP was recently shown to interact with both p53 and HDM2, inhibiting auto-ubiquitination of the latter and thereby promoting p53 degradation. In this study, we further investigated the interaction between TCTP and HDM2, mapping the reciprocal binding sites of TCTP and HDM2. TCTP primarily interacts with the N-terminal, p53-binding region of HDM2 through its highly basic domain 2. Furthermore, we discovered that Nutlin-3, a small molecule known to promote apoptosis and cell cycle arrest by blocking binding between HDM2 and p53, has a similar inhibitory effect on the interaction of HDM2 and TCTP. This result may provide an additional explanation of how Nutlin-derived compounds currently in clinical trials function to promote apoptosis in cancer cells.

## Introduction

TCTP, also known as Fortilin/Histamine Releasing Factor (HRF), was first discovered over two decades ago as a growth promoting factor in Ehrlich ascites tumor [1]. Since then, a diverse range of biological functions have been attributed to the protein including essential roles in cell proliferation and growth regulation [2], [3], [4], [5], histamine releasing properties and other ‘cytokine-like’ activity [6], [7], [8], [9] and antiapoptotic activity. TCTP is overexpressed in many human cancers including prostate, liver and breast [10], [11], [12] and tumour reversion results in its downregulation [4]. TCTP's anti-apoptotic function is attributed in part to interactions with both anti-apoptopic (Mcl-1 and Bcl-xl) [13], [14] and pro-apoptopic (BAX) [15] members of the Bcl-2 family. Additionally, TCTP has been ascribed a role in DNA damage sensing and repair, forming complexes with ATM and the DNA binding subunits Ku70 and Ku80 of DNA-dependent protein kinase [16]. More recently, TCTP has been shown to bind directly to p53, with TCTP overexpression increasing p53 degradation and promoting lung cancer cell survival [17]. Amson *et al* have recently demonstrated binding between TCTP and the E3 ubiquitin ligase HDM2 [18]. This interaction appears to control p53 levels by inhibiting HDM2 auto-ubiquitination, thereby promoting p53 ubiquitination and degradation.

In this study, we mapped the TCTP binding site to the N-terminal, p53-binding domain of HDM2, and found that mutations in the HDM2 α2 helix forming part of the p53 binding cleft significantly compromise binding. The HDM2 binding site on TCTP was also mapped to the basic domain 2 of TCTP, with residues 80–133 playing a crucial role in the interaction.

Nutlin-3 is a small molecule which binds to the p53 binding pocket of HDM2, thereby inhibiting wild type p53-HDM2 interaction, attenuating p53 degradation and activating cell cycle arrest/apoptosis mediated by the p53 network [19]. We further demonstrate that Nutlin-3 inhibits the TCTP-HDM2 interaction both in vitro and ex vivo, thus highlighting an additional mechanism through which Nutlin-3 abrogates HDM2 function.

## Materials and Methods

### Reagents

DO1 antibody was a kind gift from Dr Borivoj Vojtesek. Anti-FLAG and anti-HA antibodies were from Sigma. Nutlin-3 was from Calbiochem. The following oligonucleotides (FBCO) were used:

1)TCTP-F: 5′-ATGATTATCTACCGGGACCTCA-3′

2)TCTP-R: //5′-TTAACATTTTTCCATTTCTAAACCATCC-3′

3)TCTPINF-F: 5′- AAGGAGATATACATATGATTATCTACCGGGACCTCATC -3′

4)TCTPFLAGINFR: 5′-GGTGGTGGTGCTCGAGTTATTTATCATCATCATCTTTATAATCACATTTT

TCCATTTCTAAACCATCC -3′

5) TCTPinf3.1HIND-F: 5′- GCGTTTAAACTTAAGCTTACCATGATTATCTACCG

GGACCTCATC -3′

6) TCTPinf3.1FLAG-R: 5′- GGCCCTCTAGACTCGAGTCACTTGTCGTCGTCGTCC

TTGTAGTCACATTTTTCCAfTTTCTAAACCATCC -3′

7) petF2: 5′-CATCGGTGATGTCGGCGAT-3′

8) petRC: 5′-GATATAGTTCCTCCTTTCAGCA-3′

9) HDM491HA-R: 5′- CAGTTAAGCGTAATCTGGAACATCGTATGGGTAGGGGAAATAAG

TTAGCACAAT -3′

10) HDM339HA-R: 5′- CAGTTAAGCGTAATCTGGAACATCGTATGGGTACCCTTTATCTTT

CCCTTTATC -3′

11) HDM302HA-R: 5′- CAGTTAAGCGTAATCTGGAACATCGTATGGGTAGTCAGCTAAGG

AAATTTCAGG -3′

12) HDM109HA-R: 5′- CAGTTAAGCGTAATCTGGAACATCGTATGGGTATACTACCAA

GTTCCTGTAGAT -3′

13) HDM65HA-R: 5′-TTACGCATAATCCGGCACATCATACGGATAGCTTGGCACGCCAAA CAAATC-3′

14)HDM43HA-R: 5′-TTACGCATAATCCGGCACATCATACGGATAATCATATAATCGTTT AGTCAT-3′

15) TCTPFLAG133-R: 5′-TTATTTATCATCATCATCTTTATAATCAGGTTTTACTCTTTCTGG TCTCTG-3′

16) TCTPFLAG79-R: 5′-TTATTTATCATCATCATCTTTATAATCCTGCAGGTGATGGTTCAT G-3′

17)TCTPFLAG40-R: 5′-TTATTTATCATCATCATCTTTATAATCTTCTGTCCTACTGACCAT CTTCC

18)HDML54A-1:5′-CTATGAAAGAGGTTGCGTTTTATCTTGGCCAG-3′

19)HDML54A-2:5′-CTGGCCAAGATAAAACGCAACCTCTTTCATAG-3′

20)HDMY48A-1:5′-GCACAAAAAGACACTGCGACTATGAAAGAGGT-3′

21)HDMY48A-2:5′-ACCTCTTTCATAGTCGCAGTGTCTTTTTGTGC-3′

22)HDMY56A-1:5′-GAAAGAGGTTCTTTTTGCGCTTGGCCAGTATATTA-3′

23)HDMY56A-2:5′-TAATATACTGGCCAAGCGCAAAAAGAACCTCTTTC-3′

24)HDMY60A-1:5′-CTTTTTTATCTTGGCCAGGCGATTATGACTAAACG-3′

25)HDMY60A-2:5′-CGTTTAGTCATAATCGCCTGGCCAAGATAAAAAAG-3′

26)HDMM62A-1:5′-CTTGGCCAGTATATTGCGACTAAACGATTATATG-3′

27)HDMM62A-2:5′-CATATAATCGTTTAGTCGCAATATACTGGCCAAG-3′

28) HDMNtermdel-F: 5′-AAGGACCTTGTACAAGAGCTTCAGG-3′

29) petATG-R:5′-CATATGTATATCTCCTTCTTAAAGTTAAAC-3′

### Nucleic acid manipulation

The TCTP gene was amplified by reverse-transcription PCR on RNA extracted from AGS cells using primers 1 and 2, re-amplified using primers 3 and 4, and cloned into the NdeI-HindIII sited of pET22-b by infusion cloning (Clontech). The gene was then amplified with primers 5 and 6, and cloned by infusion cloning into the HindIII-Xho1 sites of pcDNA3.1a(+).

Templates for in vitro transcription/translation were prepared by PCR amplification of the respective gene cloned in pET22 vector using primers 7 and 8. C-terminal deletion templates were prepared by PCR using primer 7 along with one of primers 9–14 (for HDM2) and primers 15–17 (for TCTP). Primers 9–14 additionally encode a C-terminal HA tag. Primers 15–17 additionally encode a C-terminal FLAG tag. Quickchange mutagenesis (Stratagene) was used to mutate specific residues in TCTP to alanine using primers 18–27. HDM2Δ1-109 was made by PCR amplification of parental HDM2-pet22 plasmid using primers 28–29 followed by phosphorylation using T4 polynucleotide kinase and intramolecular ligation. Vectors for cell culture work were constructed from the parental plasmid HDM2-CMV. HDM2-M62A-CMV was constructed via Quikchange mutagenesis using primers 26 and 27.

### In vitro transcription-translation

Proteins were synthesised by in vitro transcription/translation using the PURESYSTEM kit (NEB). 10 ng of HDM2 PCR template (∼1.7 Kb) was used per 5 µL reaction. The amounts of all other templates were appropriately adjusted to maintain same molar concentration. ZnCl_2_ was added to a final concentration of 0.5 µM for expression of HDM2 and p53 proteins. p53 protein was synthesised at 30°C for 1.5 hours. All other proteins were synthesised at 37°C for 1 hour. Completed reactions were incubated on ice until required.

### Pull-down assays

Protein G beads (Invitrogen) were incubated with anti-HA antibody or anti-Flag antibody (1 µg per 5 µL beads) for 1 hour in PBST-1%(w/v)BSA and subsequently washed twice in PBST-0.1%(w/v)BSA and once in PBS to remove non-specifically bound protein. In vitro synthesised protein (5 µL per 5 µL beads) was added directly to beads and incubated on a rotator for 45 minutes. Beads were washed and incubated with in vitro extract containing second protein as before. For competition experiments, beads were incubated with Nutlin-3, p53 peptide/control peptide [20] in PBS for 45 minutes before washing and addition of second protein. Beads were finally washed as before and bound proteins eluted by resuspension in 20 µL SDS-PAGE loading buffer and incubation at 95°C for 5 minutes. Where required, blank in vitro extract (no template DNA added) was used as control.

For pull-downs using peptides the following biotinylated peptides (Mimotopes) were used:

TH2 : GGGSTSFTKE AYKKYIKDYMKSIKGKLEEQRPER

TH3: GGGSRPERVKPFMTGAAEQI KHILANFKNYQ

TH3-NL: GGGSVKPFMTGAAEQIKHILANFKNYQ

α2: GGGSAQKDTYTMKEVLFYLGQYIMTKR

GS-control : GGGSGGGSGGGSGGGSGGGS

p53 peptide: QETFSDLWKLLP

Peptides were immobilized on Dynabeads® M-280 Streptavidin beads (Life Technologies) by incubating 10 µg of each peptide with 15 µL of beads (per sample) in PBST- 3%BSA(w/v) buffer on a rotator for 2 hours at room temperature. Beads were washed twice to remove unbound peptides before incubation with either purified HDM2 (1–125)(15 µM) or TCTP (15 or 30 µM) proteins for 4 hours at 4°C on a rotator. Captured proteins were eluted as above. A separate pull-down was also performed as described above, but in the presence of either DMSO or Nutlin-3 (2/20/200 µM) during the protein-peptide incubation step.

For pull-down of purified TCTP, cobalt beads were first blocked in PBS-3%BSA(w/v) for one hour, before coating with 20 µM recombinant his-tagged HDM2 (residues 17–125) or control peptide (N-HHHHHHYPYDVPDYA-C) on a rotator at 4°C for one hour. Beads were then washed once before a 4-hour incubation at 4°C with 7 µM of TCTP protein. As a competitor, 200 µM of Nutlin-3 was added to the respective supernatant during bead coating and pull-down steps. Finally, beads were washed twice in PBST-0.1%BSA(w/v), once in PBS and proteins eluted in SDS-PAGE loading buffer prior to Western analysis.

### Protein purification

Both TCTP and HDM2 (amino acids 1–125) were expressed as GST-fusion proteins using the pGEX-6P-1 expression vector. Both proteins were initially passed through a 5 mL GSTrap™ FF (GE life sciences) column and eluted following on-column cleavage with precission protease. Protein fractions were analyzed with SDS page gel and concentrated using a Centricon (3.5 kDa MWCO) concentrator (Millipore). HDM2 protein samples were then dialyzed into a buffer solution containing 20 mM Bis-Tris, pH 6.5, 0.05 M NaCl with 1 mM DTT and loaded onto a monoS column pre-equilibrated in buffer A (20 mM Bis-Tris, pH 6.5, 1 mM DTT). Bound protein was eluted with a linear gradient of 1 M NaCL over 25 column volumes. For TCTP the same protocol was followed but buffers instead contained 20 mM Tris at pH 8.0 and the protein was loaded onto a monoQ column before being eluted. Protein fractions were identified using SDS page gel and protein concentration measured using absorbance at A_280_.

### Western blot Analysis

Immunoprecipitated proteins were subjected to electrophoresis, transferred to nitrocellulose membranes and probed for TCTP with horseradish peroxidase conjugated anti-flag antibody (Sigma) or for HDM2 with anti-HA antibody followed by rabbit anti-mouse (Dakocytomation). P53 was probed for using horseradish peroxidise conjugated DO1 antibody (Santa Cruz). For peptide-pull-down assays, TCTP was detected with anti-TCTP antibody (ab37506, Abcam) and HDM2 was detected with 4B2a anti-HDM2 antibody.

### Fluorescence polarization (FP)

Fluorescence polarization measurements were performed using purified HDM2 (1–125) protein and carboxyfluorescein (FAM) labeled 12-1 peptide (FAM-RFMDYWEGL-NH_2_) on the EnVision™ Plate Reader (Perkin Elmer). Competition measurements were carried out in triplicate, containing 50 nM of fluorescence peptide, with or without 250 nM of HDM2 (1–125) and the respective competitors (TH2, TH3, Nutlin-3, p53 peptide or GS-control peptide) in 50 µL of PBS-0.005%(v/v)Tween-20 buffer

### Cell culture

H1299 p53^−/−^ cells [21] were maintained in Dulbecco's modified Eagle's medium (DMEM) with 10% (v/v) foetal calf serum (FCS) and 1% (v/v) penicillin/streptomycin. The cells were seeded at 1.4×10^5^ cells/well in 6-well plates, 24 hours prior to transfection. A total of 1.375 µg of expression construct DNA was transfected per well with lipofectamine (Invitrogen) according to the manufacturer's instructions. MG132 (Calbiochem) was also added at a final concentration of 20 µM 4.5 hours post-transfection to prevent proteasomal degradation. 10 µM Nutlin-3 was added to selected wells 4.5 hours post-transfection. HCT116 p53^−/−^ cells [22] were maintained in McCoy's 5A medium with 10% (v/v) foetal calf serum (FCS) and 1% (v/v) penicillin/streptomycin. The cells were seeded at 2.8×10^6^ cells per 10 cm^2^ dish and 10 µM Nutlin-3 was added to selected dishes 24 hours post seeding. Drug treatment was allowed to proceed for 1 hour prior to harvesting.

### Immunoprecipitation and western blot analysis

H1299 p53^−/−^ cells were harvested 24 hours after transfection and lysed with lysis solution (Applied Biosystems) supplemented with both protease and phosphatase inhibitors. 10 µL of anti-HA (Sigma) antibody-coated protein G Dynabeads (Invitrogen) was used per reaction. Beads were washed twice in PBS with 0.1% (v/v) Tween-20 and incubated with 150 µg of cell lysate on a rotator at 4°C for 3 hours before washing three times with PBS with 0.1% (v/v) Tween-20. The beads were resuspended in 20 µL of SDS-PAGE loading buffer and the protein complexes eluted by incubation at 95°C for 5 mins. HCT116 p53^−/−^ cells were harvested 1 hour post drug treatment and lysed with modified RIPA buffer (50 mM Tris-HCl pH 7.4–8.0, 150 mM NaCl, 1% NP-40). Beads were prepared as above and incubated with 1 µg cell lysate at 4°C overnight with 2 µg of 2A9 antibody (Abcam). The beads were then washed as described for H1299 p53^−/−^ cells and the protein complexes eluted by incubation at 95°C for 5 mins. Immunoblotting was carried out with the relevant antibodies and identified by Immun-star™ westernC™ kit (Bio-rad). 5 µg of H1299 p53^−/−^ cell lysate and 20 µg of HCT116 p53^−/−^ cell lysate per reaction was also used to check expression levels of relevant proteins via western blot.

## Results

**Figure 1:**
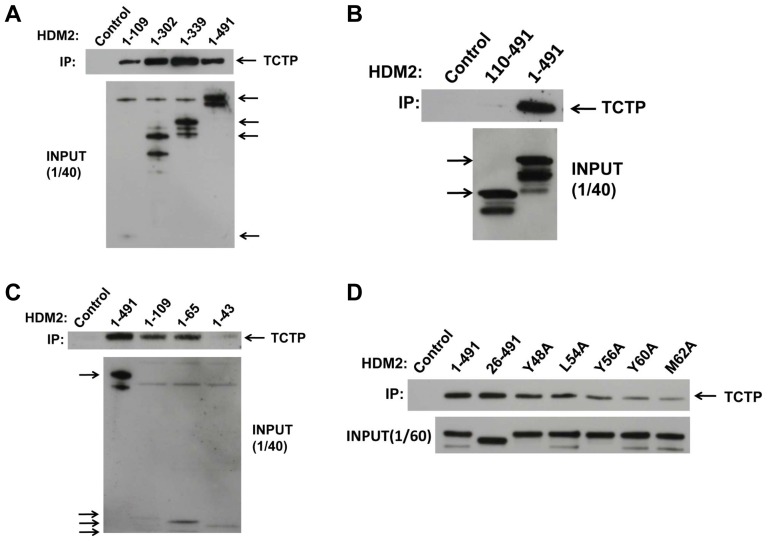
TCTP interacts with the N-terminal region of HDM2. A, in vitro pull-down of TCTP by C-terminally truncated HDM2 variants. Upper panel, Western blot of pulled down TCTP (anti-FLAG antibody). Lower panel, input levels of respective HDM2 variants (anti-HA antibody, arrowed). B, in vitro pull-down of TCTP by HDM2 (110–491). Upper panel, Western blot of pulled down TCTP. Lower panel, input levels of respective HDM2 variants (arrowed). C, in vitro pull-down by HDM2 N-terminal domain deletion mutants. Upper panel, Western blot of pulled down TCTP. Lower panel, input levels of respective HDM2 variants (arrowed). Control lanes indicate TCTP pull-down in the absence of HDM2. D, in vitro pull-down of TCTP by HDM2 with point mutations in α2 helix. Upper panel, Western blot of pulled down TCTP (anti-FLAG antibody) by indicated mutants. Lower panel, input levels of respective HDM2 proteins.

**Figure 2:**
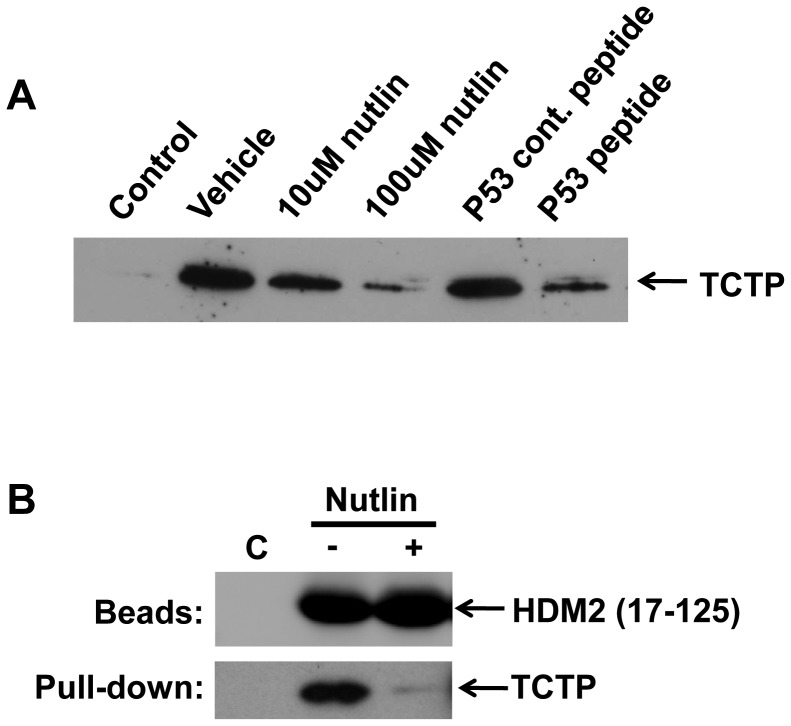
Inhibition of TCTP-HDM2 interaction by Nutlin-3 and p53 peptide. A, In vitro translated HDM2 was immobilised on beads and pre-treated with indicated amounts of Nutlin-3 or p53 peptide/p53 control peptide (1 mM). Bound TCTP detected by Western blot (anti-FLAG antibody). Control lane indicates TCTP pull-down in the absence of HDM2. B, Recombinant HDM2 (residues 17–125) or control peptide (GS-control) was immobilised on beads and incubated with recombinant TCTP either in presence or absence of 200 µM Nutlin-3.

We first carried out pull-down assays using in vitro expressed proteins to map the interaction site(s) between TCTP and HDM2. HDM2 (tagged at the C terminus with HA) was bound to protein G beads coated with anti-HA antibody. The beads were subsequently incubated with TCTP (FLAG-tagged). Bound TCTP was identified via Western blot. C-terminal deletion analysis of HDM2 indicated the N-terminal region alone (residues 1–83) was sufficient for interaction with TCTP (Figure 1A). Notably, when compared to full-length HDM2, deletion of C-terminal residues 303–491 (containing the zinc finger and RING domains) and 340–491 (containing the RING domain) led to increased interaction with TCTP. Deletion of residues 1–109 in HDM2 resulted in very minimal interaction with TCTP (Figure 1B), suggesting a predominant N-terminal interaction site. Further deletion analysis highlighted the importance of residues 44–65 in the interaction (Figure 1C). This region comprises the α2 helix that forms part of the p53 binding cleft of HDM2 [23]. Alanine scanning of this region was carried out to further map the interaction. Residues Y48, L54, Y56, Y60 and M62 were individually mutated to alanine in full length HDM2 and the interaction with TCTP assayed. The results show a progressive reduction in the interaction with TCTP as residues along the α2 helix are mutated, with the M62A mutant showing considerably weaker binding (Figure 1D). We additionally deleted the first 25 amino acids in HDM2 comprising the flexible lid region [24]. Binding to TCTP was unperturbed, further confirming the importance of residues 44–65 in the interaction with TCTP (Figure 1D). M62 forms part of the binding pocket that accommodates the side chain of F19 in the p53 transactivation domain [25]. We therefore investigated whether the TCTP binding site of HDM2 overlapped with the p53 binding site. HDM2 was first incubated with a p53 peptide corresponding to residues 19–26 of the p53 transactivation domain that interact with HDM2 [25], followed by TCTP. We also pre-incubated with the HDM2 inhibitor Nutlin-3, which binds to the p53-binding cleft [19]. p53 peptide, but not control peptide (p53 peptide with critical contact residues F19, W23, L26 mutated to alanine) diminished TCTP binding (Figure 2A). Nutlin-3 also showed a dose responsive reduction in TCTP binding. Inhibition by Nutlin-3 was again observed when recombinant HDM2 N-terminal domain was used to pull down recombinant TCTP (Figure 2B).

**Figure 3:**
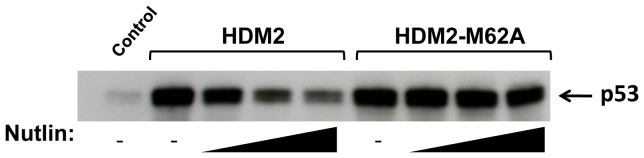
Nutlin-3 does not inhibit p53 binding to HDM2-M62A. In vitro translated HDM2 or HDM2-M62A was immobilised on beads and pre-treated with 0/100/200/400 µM Nutlin-3 prior to incubation with in vitro translated p53. Bound p53 detected by Western blot using DO1 anti-p53 antibody. Control lane indicates p53 pull-down in the absence of HDM2.

We further investigated the HDM2-M62A mutant to see if it retained the capacity to bind p53. As shown in Figure 3, it bound to p53 as strongly as wild-type, indicating the lack of any major structural perturbation due to this mutation. However, whilst Nutlin-3 showed a dose-responsive knock down in the HDM2-p53 interaction, the M62A mutant proved recalcitrant to Nutlin-3 inhibition.

**Figure 4:**
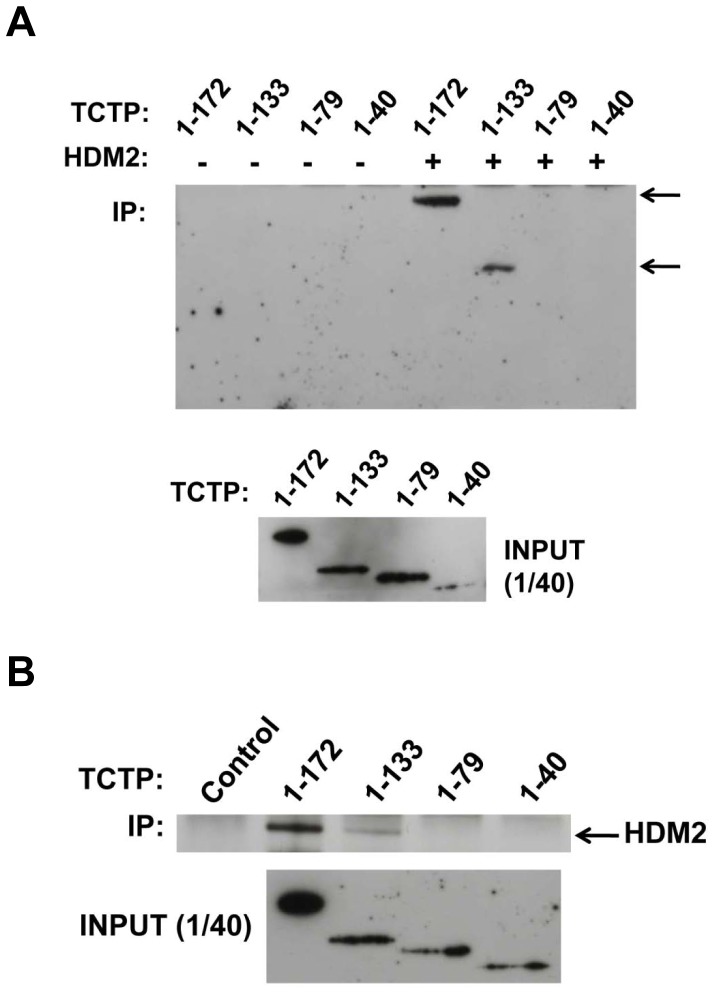
Mapping of the TCTP interaction site with HDM2. A, in vitro precipitation of C-terminally deleted TCTP mutants by HDM2. Upper panel, Western blot of pulled down TCTP variants (anti-FLAG antibody). Lower panel, input levels of respective TCTP variants. Control lanes indicate pull-down of TCTP variants in absence of HDM2. B, Reciprocal pull-down of HDM2 by C-terminally deleted TCTP mutants. Upper panel, Western blot of pulled down HDM2 (anti-HA antibody). Lower panel, input levels of respective TCTP variants.

**Figure 5:**
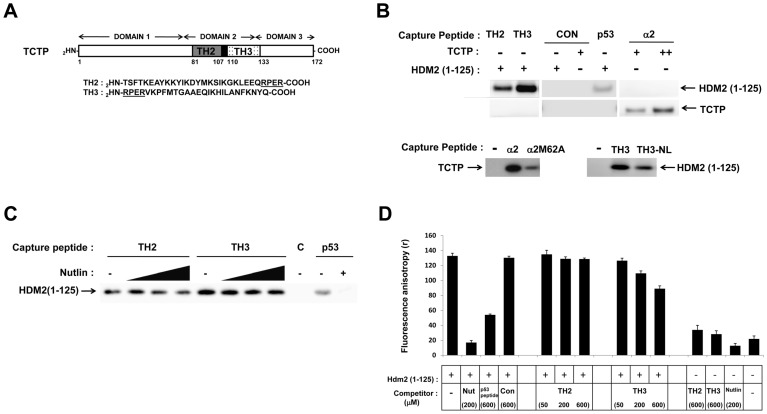
Interaction of peptides with recombinant HDM2 (1–125) and TCTP. A, Schematic depicting relative positions and sequences of TH2 peptide (grey box), overlap region (black box, underlined in amino acid sequences) and TH3 peptide (stippled box) comprising the helix-loop-helix motif in the highly basic domain 2 of TCTP. B, Western blot showing binding of recombinant HDM2 (1–125) or TCTP to beads pre-coated with TCTP peptides (TH2, TH3) or HDM2 peptide (α2) respectively (top panel). Control pull-down (CON) was performed using non-specific peptide (tandem GSSS 20-mer peptide). Positive control (DO1) carried out using P53 peptide to pull down HDM2 (1–125). Bottom left panel shows binding of TCTP to immobilised α2 and α2-M62A peptides. Bottom right panel shows binding of HDM2 (1–125) to immobilised TH3 and TH3-NL peptides. C, Effect of Nutlin-3 (2/20/200 µM) on binding of TH2 and TH3 peptides to HDM2 (1–125). Lane C indicates HDM2 binding to non-specific peptide. p53 peptide used as positive control in absence or presence of Nutlin-3 (200 µM). D, Fluorescence polarization assay measuring displacement of FAM-labeled p53 peptide (12.1) from HDM2 (1–125) by TH2/TH3 peptides (50/200/600 µM), control peptide (600 µM) and Nutlin-3 (200 µM).

We additionally carried out C-terminal deletion analysis of TCTP to map its interaction site with HDM2. Whilst full-length TCTP and residues 1–133 bind to HDM2, further truncation to 79 residues completely ablates HDM2 binding (Figure 4A). The same result was obtained with the reverse configuration of the IP (TCTP captured on beads used to pull down HDM2, Figure 4B). This indicated a probable interaction interface within amino acids 80–133 of TCTP which comprises an helix-loop-helix motif. We next carried out a series of pull-down experiments using 2 synthetic peptides spanning this region of TCTP (TH2: residues 81–110; TH3: residues 107–133) (Figure 5A) along with a peptide spanning the HDM2 α2 helix (residues 43–65). The results in Figure 5B (top panel) indicate that both TH2 and TH3 peptides immobilised on beads can pull down recombinant HDM2 N-terminal domain (residues 1–125), with TH3 showing a stronger binding phenotype. Additionally, immobilised HDM2 α2 helix peptide pulled down recombinant full-length TCTP (bottom left panel). Strikingly, the same peptide with the M62A mutation (α2M62A) showed significantly reduced pull-down of TCTP. Neither recombinant TCTP nor HDM2 (1–125) bound to an immobilised control peptide (CON, top panel). As a positive control we used the p53 peptide known to interact with the N-terminal domain of HDM2. Peptides TH2 and TH3 share the sequence RPER comprising the loop region (residues 107 to 110) within the helix-loop-helix motif defining the TCTP basic domain 2 (residues 80–133). TH3 peptide lacking this sequence (TH3-NL) showed significantly reduced pull-down of HDM2 (1–125) (Figure 5B, bottom right panel), highlighting the important contribution of this region to the interaction. The pull-down experiments with TH2 and TH3 peptides were next repeated in the presence of Nutlin-3 (Figure 5C). Interaction of TH2 peptide with HDM2 (1–125) was clearly perturbed in a dose-responsive manner. The TH3 peptide interaction was minimally inhibited at the highest concentration of Nutlin-3 used, consistent with its stronger binding phenotype. This was also observed when the interactions were assayed by fluorescence polarisation (Figure 5D). HDM2 (1–125) was pre-incubated with fluorescently labelled p53 peptide and the ability of the TCTP peptides to displace this was measured. TH3 peptide, but not TH2 was able to displace the p53 peptide, although to a lower extent than the positive controls Nutlin-3 and un-labelled p53 peptide.

**Figure 6:**
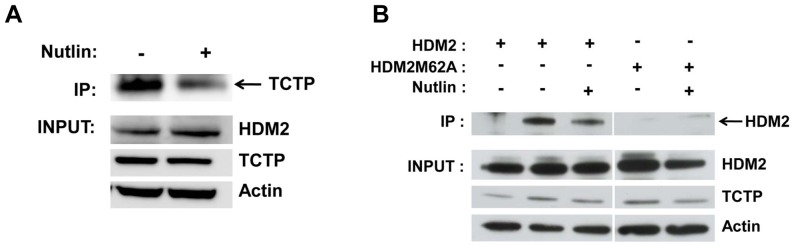
Disruption of HDM2-TCTP interaction by Nutlin-3 in HCT116 cells. A, Co-IP of endogenous HDM2-TCTP complexes in HCT116 p53^−/−^ cells. Upper panel, Western blot of immunoprecipitated TCTP in absence or presence of Nutlin-3 (10 µM). Lower panels, input levels of HDM2 and TCTP. B, Co-IP of exogenous HDM2 and HDM2-M62A with endogenous TCTP in H1299 p53^−/−^ cells. Upper panel, Western blot of immunoprecipitated HDM2 variants. First lane indicates control co-IP using non-specific antibody on beads. Lower panels, input levels of respective HDM2 variants and TCTP.

We next investigated the effect of Nutlin-3 on the endogenous TCTP-HDM2 interaction in the HCT116 p53^−/−^ cell line. Co-immunoprecipitation was carried out using anti-HDM2 antibody to capture TCTP-HDM2 complexes. The results (Figure 6A) indicate disruption of the TCTP-HDM2 interaction by Nutlin-3, consistent with the previous in vitro data (Figures 2, 5C). The same phenotype was seen using exogenously expressed HDM2 in the p53-null H1299 cell line (Figure 6B). Furthermore, the HDM2-M62A mutant showed very weak interaction with TCTP compared to wild-type, again consistent with the in vitro result (Figure 1D).

## Discussion

It was recently shown that TCTP increased MDM2-mediated ubiquitination of p53, and that this effect was inhibited by Nutlin-3 [18]. In the present study, we provide a possible mechanistic rationale for this observation by showing that TCTP and Nutlin-3 can compete for binding to the p53-binding cleft in the N-terminus of HDM2. The p53-binding cleft consists of 4 α helices and a pair of β sheets cap each end [25]. Deletion analysis implicated the α2 helix forming one side of the cleft as contributing significantly to the TCTP interaction site. Alanine scanning of the α2 helix further identified critical residues involved in the interaction, with M62 being of particular importance. This residue comprises part of the binding pocket that accommodates F19 of p53 and the ethyl ether moiety of Nutlin-3 [19]. Notably, binding of p53 to HDM2-M62A was not inhibited by Nutlin-3, suggesting against mutation-induced structural deformation. Based on these observations, we propose a model wherein TCTP binds a sub-region of the p53-binding cleft to exert its chaperone-like function on HDM2. TCTP is subsequently displaced by p53 due to its higher affinity for the p53-binding cleft. Additionally, a secondary p53 interaction site within the acidic domain of HDM2 [26], [27] may contribute towards high affinity interaction and TCTP displacement.

A highly allosteric model of HDM2 function has emerged, wherein conformational changes within structurally discrete domains impact on its interaction with p53 [28]. Notably, the C-terminal RING finger domain (residues 438 to 479) regulates the binding affinity of the N-terminal region to p53, and mutations in this domain have also been shown to modulate Nutlin-3 efficacy [29]. Our results indicate increased interaction of HDM2 with TCTP when the C-terminal zinc finger (residues 300 to 332) and/or RING domains were deleted. Allosteric modulation by these domains may therefore also regulate the HDM2-TCTP interaction.

We additionally mapped the TCTP interaction site to within residues 80–133 corresponding to the basic domain 2. This region comprises an helix-loop-helix motif [30] and our data show residues within the loop to contribute significantly to the interaction with HDM2. Domain 2 has been implicated in TCTP's interaction with tubulin [2], calcium [31], and the Na,K-ATPase α subunit [32]. Furthermore, TCTP has recently been shown to interact with p53 through either domain 2 [17] or N- and C-terminal regions [33]. We note that Amsen et al have mapped an interaction interface between residues 1–68 of TCTP and residues 302–435 of HDM2 using SPR and recombinant proteins [18]. This interaction site was not evident in our results using pull-down assays with in vitro expressed proteins. We are presently carrying out further work to evaluate the contribution of this additional binding interface to the overall TCTP-HDM2 interaction both in vitro and ex vivo.

**Figure 7:**
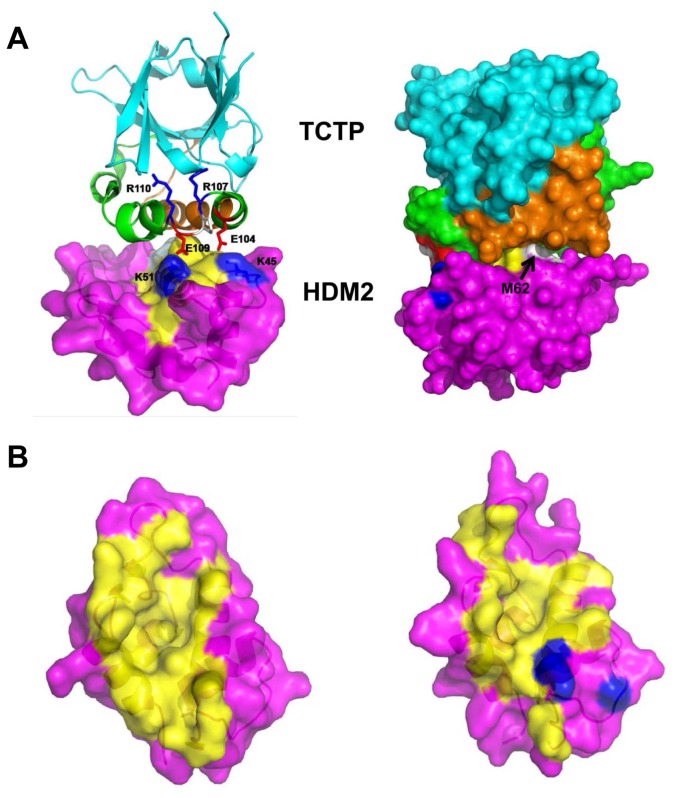
A model of the putative interactions between the N-terminal domain of HDM2 and TCTP. Model was constructed by docking the structures of the two proteins (for HDM2, the N-terminal domain consisting of residues 25–109, RCSB entry 1YCR [25]; for TCTP, residues 1–172, RCSB entry 1YZ1). The two proteins were aligned in different orientations that suggested the experimentally observed interactions between TH2 and TH3 and the p53-binding cavity of HDM2. Molecular dynamics (MD) simulations were carried out for up to 200 ns as previously described [34]. The simulations yielded only one complex as stable and a snapshot taken from this simulation is shown (details will be published elsewhere). HDM2 is depicted in magenta with the α2 helix (residues 44–65) highlighted in yellow. The TH2 and TH3 peptide sequence of TCTP are respectively highlighted in brown and green. A, Left: the interactions between the RPER loop of TCTP and the N-terminal domain of MDM2; Right: the TCTP-MDM2 complex rotated by 90° to show the buried and packed M62 (in white spheres); the patches in blue, red and white to the left are the same as in left depiction but with the sidechains not highlighted for clarity. B, Left: top view of the residues on the HDM2 surface (magenta) that interact (yellow) with p53. Right: top view of residues on the HDM2 surface (magenta) that interact (yellow) with TCTP. K45 and K51 are depicted in blue.

Using molecular simulations, a docked complex of TCTP with HDM2 (1–125) was derived (Figure 7). Stable interactions of the TH2 and TH3 helices of TCTP with residues in the HDM2 nutlin-binding pocket were observed in accordance with the alanine scanning data (Figure 1D). The RPER loop region (residues 107–110) connecting TH2 and TH3 is stabilized by intramolecular interactions of R107 and R110 with residues in TCTP, whilst the backbone carbonyl of P108 and the side chain of E109 are stabilized by K51 of HDM2. Additionally, E104 of TCTP is also stabilized by K45 of MDM2 (Figure 7A). The loss of affinity seen when the loop region was deleted from peptide TH3 (Figure 5B) could result from removal of one salt bridge and/or significant perturbation of the other. It is clear from Figure 7A that the α2 helix of HDM2 interacts with both TH2 andTH3 of TCTP, with M62 closely packed under TH3.

TCTP has been shown to be significantly upregulated in a number of human cancers, with high levels of TCTP correlating with poor prognosis [10], [11] [12], [18]. Nutlin-3 has been shown to be most effective in cancers which express wild type p53 and high levels of HDM2. Investigation of the effects of Nutlin-3 in cancer cells and animal models with high levels of TCTP overexpression may prove valuable.

The value of inhibiting the p53-HDM2 interaction as a possible target for cancer therapeutics is currently an area of great activity. The discovery that TCTP not only interacts with both these proteins, but has a binding site on HDM2 which overlaps with that of p53, adds further complexity to the p53-HDM2 interaction model. Further work must be done to understand the cellular implications of these interactions and their consequences for therapies aimed at inhibiting p53-HDM2 binding.
